# Synthesis and Photothermal Effects of Intracellular Aggregating Nanodrugs Targeting Nasopharyngeal Carcinoma

**DOI:** 10.3389/fbioe.2021.730925

**Published:** 2021-09-16

**Authors:** Ying Zhong, Naveen Kumar Bejjanki, Xiangwan Miao, Huanhuan Weng, Quanming Li, Juan Zhang, Tao Liu, Raghu Vannam, Minqiang Xie

**Affiliations:** ^1^ Department of Otolaryngology-Head and Neck Surgery, Zhujiang Hospital, Southern Medical University, Guangzhou, China; ^2^ Department of Otolaryngology-Head and Neck Surgery, Zhuhai People’s Hospital, Zhuhai, China; ^3^ Department of Thyroid Surgery, Shantou Central Hospital, Shantou, China; ^4^ Department of Otolaryngology-Head and Neck Surgery, Guangdong Provincial People’s Hospital, Guangdong Academy of Medical Sciences, Guangzhou, China; ^5^ Piramal Pharma Solutions, Riverview, MI, United States

**Keywords:** folic acid targeting, intracellular aggregation, indocyanine green, photothermal effect, nasopharyngeal carcinoma

## Abstract

Chemotherapy for the treatment of nasopharyngeal carcinoma (NPC) is usually associated with many side effects; therefore, its treatment options have not yet been completely resolved. Improving distribution to the targeted tumor region and enhancing the cellular uptake of drugs can efficiently alleviate the above adverse medical effects. Near-infrared (NIR) laser light-mediated photothermal therapy (PTT) and photodynamic therapy (PDT) are promising strategies for cancer treatment. In the present study, we developed an efficient multifunctional nanocluster with enhanced targeting and aggregation efficiency for PTT and PDT that is composed of a biocompatible folic acid (FA), indocyanine green (ICG) and 2-cyanobenzothiazole (CBT)-functionalized peptide labeled with an aldehyde sodium alginate-modified magnetic iron oxide nanoparticle (ASA-MNP)-based nanocarrier. FA can bind to folate receptors on cancer cell membranes to enhance nanocluster uptake. CBT-modified peptide can react with glutathione (GSH), which is typically present at higher levels in cancer cells, to form intracellular aggregates and increase the local concentration of the nanodrug. In *in vitro* studies, these nanodrugs displayed the desired uptake capacity by NPC cells and the ability to suppress the growth of cancer cells under laser irradiation. Animal studies validated that these nanodrugs are safe and nontoxic, efficiently accumulate in NPC tumor sites following injection *via* the caudal vein, and shows superior inhibition of tumor growth in a tumor-bearing mouse model upon near-infrared laser irradiation. The results indicate the potential application of the multifunctional nanoparticles (NPs), which can be used as a new method for the treatment of folate receptor-positive NPC.

## Introduction

Nasopharyngeal carcinoma (NPC), arising from nasopharyngeal epithelial tissues, is the most common malignant tumor in the head and neck and is prevalent in southern China (Simo et al., 2016). Platinum-based chemotherapy and chemoradiotherapy play a key role in the current treatment of NPC (Liu et al., 2018). However, cisplatin resistance (Weng et al., 2019), a lack of target recognition, and off-target toxicity (Braun et al., 2018)limit the therapeutic effects of the existing treatments (Wu et al., 2013; Fan et al., 2015). Therefore, novel therapeutic strategies for NPC still need to be explored to overcome these limitations and improve the efficiency of the current methods.

The biocompatible 2-cyanobenzothiazole-cysteine (CBT-Cys) click reaction has been widely used in medicinal chemistry owing to its prominent specificity, superior quantitative product production, and high fidelity in the physiological environment (Zheng et al., 2017), in which *in situ* condensation occurs between the cyano group of 2-cyanobenzothiazole (CBT) and l,2-aminothiol (Liang et al., 2011; Hai et al., 2019) under a controlled pH, disulfide reduction or enzymatic cleavage.

The intracellular cross-linked polymerization of Fe_3_O_4_@1NPs was previously reported by Yuan et al. (2016). In this polymerization, glutathione (GSH) in cancer cells can reduce cysteine disulfide bonds, enabling the condensation of the cyano group of CBT and free 1,2-aminothiol on Cys, leading to the cross-linked polymerization of Fe_3_O_4_ NPs. GSH can also be involved in the click reaction of CBT-Cys and could be applied to synthesize large NPs to achieve specific functions. Previous studies have suggested that commonly used chemotherapy drugs or small NPs can easily cause membrane leakage from cancer cells, leading to exocytosis (Serda et al., 2010). In addition, larger NPs tend to be released at a slower rate and in lower quantities than smaller NPs (Kim et al., 2015). Inspired by the theoretical and experimental studies above, we tried to employ a GSH-instructed condensation reaction and properly designed an aggregating polypeptide, Cys (StBu)-Lys-CBT, which could allow the efficient accumulation of nanodrugs at tumor sites and reduce leakage by forming large particles.

Researchers have conducted many studies on new avenues for cancer therapy. Among them, photodynamic therapy (PDT) and photothermal therapy (PTT) have been continually conducted worldwide (Apalla et al., 2010; Aniogo et al., 2017; Rao et al., 2018; Shi et al., 2018; Nakayama et al., 2020). As an important PDT and PTT photothermal agent, indocyanine green (ICG) is widely used in the synthesis of functional materials for clinical diagnosis and treatment due to its near-infrared (NIR) light absorption, amphiphilic structure and low toxicity (Wang et al., 2016; Yan et al., 2016). Recently, ICG has been integrated with different anticancer agents using nanoparticles for achieving better efficacy *via* combinational treatments (Jin et al., 2020; Liu et al., 2021; Wang et al., 2021). The irradiated ICG acts as an exogenous energy absorber to convert light energy into heat energy to kill tumors (Melamed et al., 2015), known as the PTT. At the same time, it is stimulated by NIR light to release reactive oxygen species (ROS), which react with adjacent biomolecules to cause cell death, known as the PDT. All of these studies without exception try to design nanosystems for combined photochemotherapy of cancers at very low therapeutic doses. However, drawbacks, such as water instability, photodegradability, thermal degradability, and easy lipoprotein binding, lead to the rapid removal of ICG from plasma, which limits its applications in PDT and PTT. Hence, how to improve the effective delivery of nanoparticles to tumor is absolutely necessary. Various studies have attempted to modify ICG using nanotechnology to prolong its retention time in tumors and enhance tumor suppression (Bahmani et al., 2013; Hannah et al., 2014). Folic acid (FA) has been known to be an effective ligand for targeted cancer therapies, because it binds to a tumor-associated antigen called the folate receptor (FR) (Cheng et al., 2017; Li et al., 2018; Chen et al., 2019). Our previous studies confirmed that the expression of the FR on the surface of NPC cells is much higher than that on the surface of normal cells (Xie et al., 2013). Therefore, the surface modification of nanoparticles with FA can increase their accumulation in tumors. In addition, polyethylene glycol (PEG) can be used to modify nanoparticles, prevent them from being recognized by the immune system, prolong their retention time in blood and promote the enhanced permeability and retention effect (Harris and Chess, 2003; Qi et al., 2021).

The Fe_3_O_4_ NPs have been approved as safe clinical agents and widely employed in biomedical research because of their good therapeutic effect, drug delivery and magnetic resonance imaging ability (Casula et al., 2006; Kwon et al., 2007; Vazquez-Prada et al., 2021). As is known, magnetic hyperthermia (MHT) is based on the accumulation of magnetic particles in the target site and the application of an alternating magnetic field of sufficient strength and frequency to induce nanoparticle heating (Mejias et al., 2019). As the cancer cells are considered more susceptible to hyperthermia than healthy cells, it can achieve good therapeutic effect in target site, without affecting the normal tissues. Another study found that heat can be generated rapidly in micelles containing Fe_3_O_4_ NPs by NIR irradiation (Guangzhen et al., 2012). Based on the above researches, Fe3O4 NPs were used as the medium for heat production in PTT and MHT in our study. Among them, the photothermal effect of nano-drugs is our main research content, and MHT is taken as the control group to explore more possibilities of the treatment. We took Fe_3_O_4_ NPs as cores, and modified them by complex to make them biocompatible, which is suitable for further functionalization by attaching various bioactive molecules.

The aims of the present study were the: 1) modification of the superparamagnetic iron oxide nanoparticles (SPIONs) with FA, PEG, ICG and CBT; 2) enhancement of NPC tumor suppression by the use of FA- and PEG-doped, ICG- and CBT-functionalized SPION nanoparticles (FA-PEG/CBT@SPION-ICG) combining with PTT/PDT or MHT, and preliminary investigation on anti-tumor mechanism of it; 3) clarification of the location of FA-PEG/CBT@SPION-ICG within tumor cells and the distribution *in vivo* by transmission electron microscopy (TEM) and fluorescence imaging analyses; 4) comparison of the efficacy of nanoparticles in PDT/PTT and MHT; meanwhile, evaluation of the therapeutic effect of PTT/PDT with the application of FA-PEG/CBT@SPION-ICG and a preliminary exploration of the safe laser power; 5) assessment of the hepatotoxicity and nephrotoxicity of FA-PEG/CBT@SPION-ICG.

**FIGURE 1:**
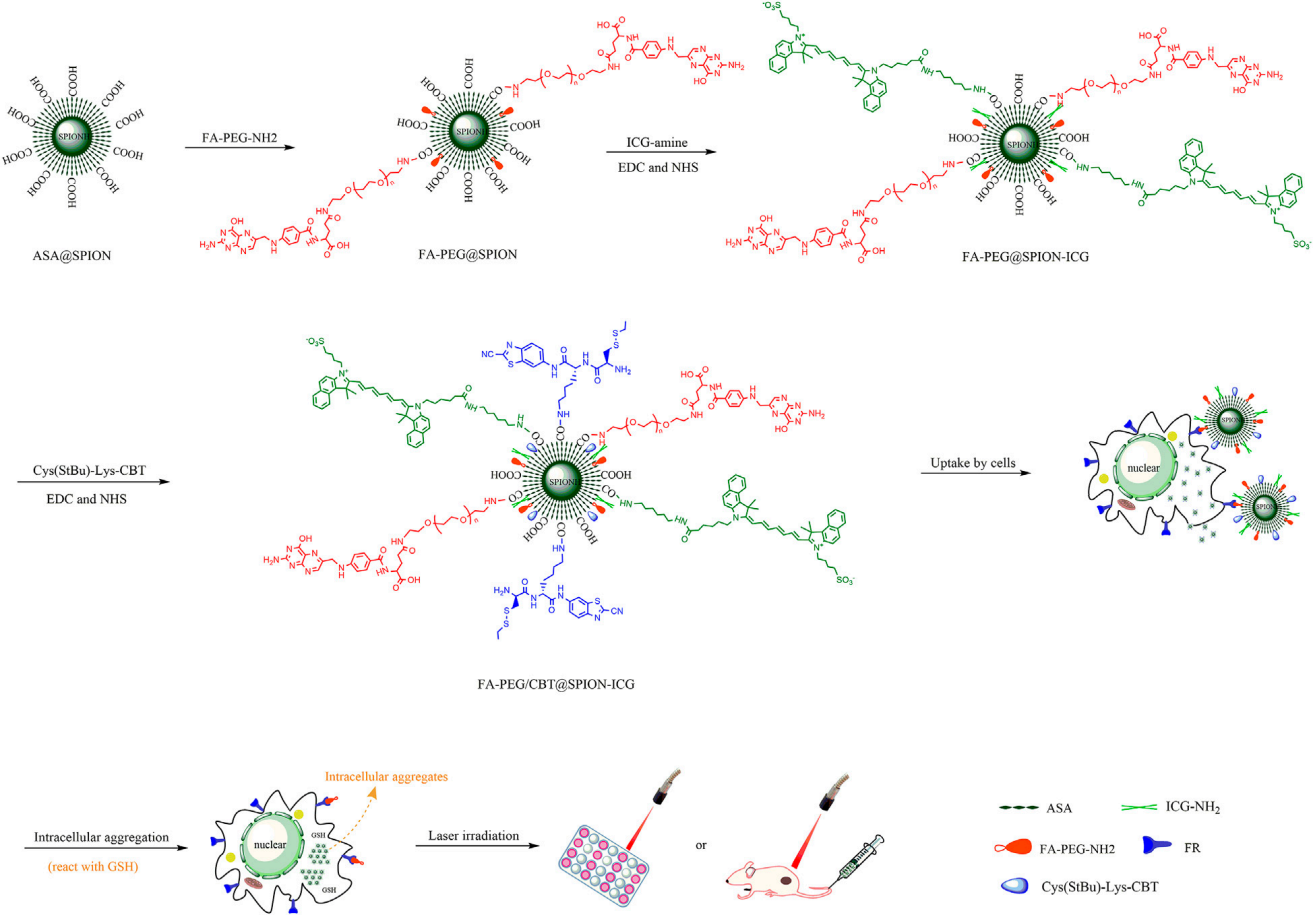
Schematic illustration of the construction of FA-PEG/CBT@SPION-ICG and photothermal therapy of nasopharyngeal carcinoma.

In this study, we developed a novel multifunctional nanomedicine, FA-PEG/CBT@SPION-ICG, with the simultaneous incorporation of an FA targeting moiety, intracellular aggregates, and photothermal agents for *in vitro*/*in vivo* tumor treatment (Figure 1). Herein, a programmed method for fabricating the nanodrug FA-PEG/CBT@SPION-ICG was developed. The therapeutic effects of this nanodrug on photothermal and photodynamic destruction, fluorescence visualization, biosafety, metabolic processes and possible mechanism were preliminarily explored. There have not been many studies in this field especially on NPC for now. These results indicate the potential benefits of FA-PEG/CBT@SPION-ICG combining with 808 nm NIR in the development of new tumor therapies.

## Materials and Methods

### Materials

FA was purchased from Genview (United States). NH_2_-PEG-NH_2_ was purchased from Yare (Shanghai, China). 1-(3-Dimethylaminopropyl)-3-ethylcarbodiimide (EDC) was purchased from purchased from Macklin (Shanghai, China). N-Hydroxysuccinimide (NHS) and CBT were purchased from Bide (Shanghai, China). ICG-amine was purchased from Xi’an Dianhua Biological Technology Co., Ltd. (Xi’an, China). Dimethyl sulfoxide (DMSO) was purchased from Meryer (Shanghai, China).

### Cell and Animal Models

The human NPC cell line HNE-1 (expressing the folate receptor) and cell lines 5–8 F (not expressing the folate receptor) were used in this study. The cells were cultured in complete growth medium (RPMI-1640 supplemented with 1% penicillin/streptomycin and 10% fetal bovine serum) at 37°C in a 5% CO_2_ atmosphere. Female BALB/c mice were obtained from the Guangdong Provincial Experimental Animal Center and were used in accordance with approved institutional protocols established by the ethics committee of Zhujiang Hospital of Southern Medical University.

### Preparation of FA-PEG/CBT@SPION-ICG

#### Synthesis of FA-PEG@SPION

Hydrosoluble Fe_3_O_4_ MNPs were prepared by the chemical coprecipitation method and then modified by aldehyde sodium alginate (ASA) according to the method reported by our team (Xie et al., 2012)^,^ based on the study of Laurienzo et al. (2005). Additionally, FA-PEG-NH_2_ was formed through the interaction of the activated carboxyl groups of FA and the amino groups of NH_2_-PEG-NH_2_. Next, the free amine group of FA-PEG-NH_2_ was conjugated with the aldehyde group on the surface of ASA@SPION *via* a Schiff base reaction. Later, NaBH_4_ was added to the above reaction system and stirred overnight to generate FA-PEG@SPION.

#### Synthesis of FA-PEG@SPION-ICG

EDC (239 μg) and NHS (138 μg) were added separately to the FA-PEG@SPION solution with an iron content of 30 mg. After 4 h of stirring, 500 μg of ICG-amine was added to the reaction system and stirred overnight. Subsequently, the solution was ultrafiltered (4,000 rpm, 10 min) twice to remove free ICG. The inner product in the ultrafiltration tube was FA-PEG@SPION-ICG, which was collected and kept from light for later use, while the outer substance was free ICG. The free ICG was collected and its absorbance was measured spectrophotometry, by which the ICG concentration in the FA-PEG@SPION-ICG solution could be calculated according to the ICG standard curve.

#### Synthesis of FA-PEG/CBT@SPION-ICG

First, the synthetic method of the aggregating polypeptide Cys (StBu)-Lys-CBT was based on Liang’s work (Liang et al., 2010). The steps are shown in Supplementary Figure S1, and the obtained product Cys (StBu)-Lys-CB2T was freeze-dried for later use. EDC (134 μg) and NHS (81 μg) were added to a dispersed solution of FA-PEG@SPION (pH = 6.0) with an iron content of 30 mg. After 6 h of stirring, the solution was ultrafiltered to remove the residual EDC and NHS to obtain the precipitate. The precipitate was dissolved in 10 ml of PBS (pH = 7.5), and 3 mg of Cys (StBu)-Lys-CBT was added and the mixture stirred overnight. Subsequently, the product was dialyzed for 1 day to generate purified FA-PEG/CBT@SPION. In the meantime, 112 μg EDC and 67 μg NHS were added separately to FA-PEG@SPION-ICG with an iron content of 30 mg and stirred for 6 h. Similarly, the solution was ultrafiltered, and the obtained precipitate was dissolved in 10 ml of PBS (pH = 7.5). Next, 250 μg of Cys (StBu)-Lys-CBT was added to this solution and stirred overnight. Finally, the product was dialyzed for 1 day to generate the final product FA-PEG/CBT@SPION-ICG. The detailed reaction scheme for the fabrication of the final product FA-PEG/CBT@SPION-ICG is shown in Supplementary Figure S2.

### Characterization of FA-PEG/CBT@SPION-ICG and the Other Key Products

As it performs the important function of intracellular aggregation, the synthetic product Cys (StBu)-Lys-CBT was extensively characterized by various methods. Hydrogen nuclear magnetic spectroscopy (^1^H NMR) was performed with an NMR spectrometer (Bruker, Germany). The molecular weights of Cys (StBu)-Lys-CBT and intermediates of FA-PEG/CBT@SPION-ICG were determined with a triple quadrupole liquid mass spectrometer (Thermo, United States). To identify the nanomedication dosage, absorbance values were obtained with an ultraviolet-visible spectrophotometer (LabTech, United States) to determine the ICG concentration in the NPs. The iron concentrations in the NPs in the liquid suspensions were determined by inductively coupled plasma optical emission spectrometry (Agilent, United States). A laser scattering particle size distribution analyzer (Nicomp, United States) was used to determine the hydrodynamic diameter and size distribution of the particles at room temperature (RT). The size and shape of the NPs were determined by TEM (JEM-2100F, Japan).

To examine the photothermal effects *in vitro*, solutions of ICG, FA-PEG@SPION-ICG, FA-PEG/CBT@SPION, and FA-PEG/CBT@SPION-ICG at an ICG concentration of 50 μg/ml or iron concentration of 500 μg/ml were added to separate 1 ml tubes and then exposed to an 808 nm laser at 0.98 W/cm^2^ or a magnetic field of 193 kHz, 350.4 A for 5 min. The temperatures of the solutions were recorded with an infrared thermal imager (DALI, China) every minute.

To verify the targeting of the nanomedicine, iron staining, fluorescence staining, laser confocal microscopy (Leica, Germany), and TEM (Hitachi H-7500, Japan) were used for the corresponding experimental study.

### *In Vitro* Cellular Uptake

HNE-1 cells and 5–8 F cells were seeded onto 24-well plates and incubated with 1 ml of complete growth medium. All of the cells were then treated with the intermediate product FA-PEG/CBT@SPION at an iron concentration of 10 μg/ml and incubated for 6 h. The amount of trivalent iron in the cells was detected with a Prussian Blue Iron Stain Kit (Solarbio China) according to the manufacturer’s instructions (Figure 3A).

In addition, to indicate the targeting characteristics to HNE-1 cells of the synthetic agents with and without folate, FITC-labeled ASA-SPION, FA-PEG@SPION, and FA-PEG/CBT@SPION each with an iron concentration of 10 μg/ml were added separately to confocal dishes and cultivated with HNE-1 cells for 6 h. The uptake of the different agents was detected with a Hoechst kit (Beyotime, China). Confocal fluorescence images were captured using confocal microscopy. We used ImageJ software for semiquantitative analysis and SPSS for data processing (Figure 3B).

To further prove the uptake ability of the final product FA-PEG/CBT@SPION-ICG by HNE-1 cells, HNE-1 cells were seeded on confocal dishes and incubated with FA-PEG/CBT@SPION-ICG at an ICG concentration of 1 µg/ml for 6 h. Free ICG was the control group. Procedures similar to those described above were used for Hoechst staining, confocal microscopy observation, and data analysis (Figure 3C).

For TEM, FA-PEG@SPION-ICG and FA-PEG/CBT@SPION-ICG (ICG concentration of 0.5 μg/ml or iron concentration of 5 μg/ml) were added to 6-well plates, cultured with HNE-1 cells for 6 h, washed with PBS, and then collected into 1.5 ml Eppendorf tubes. Next, the tubes were centrifuged at 2,000 rpm for 5 min, fixed with 500 μl of 2.5% glutaraldehyde, and placed according to the conventional temperature gradient for further fixation. Finally, thin sections were cut and observed by TEM (Hitachi, Japan) (Figure 3D).

### *In Vivo* Biologic Imaging

*In vivo* imaging was performed on anesthetized healthy BALB/c nude mice bearing HNE-1 tumors that had reached a volume of 200 mm^3^ (the tumor volume was calculated by the formula V = L×W^2^/2), where V, L, and W represent the volume, length, and width of the tumor, respectively. ICG, FA-PEG@SPION-ICG, and FA-PEG/CBT@SPION-ICG (240 μg/ml ICG for each mouse, 150 μl for each injection) were intravenously injected into the tails of the mice. After 8 h, the uptake of the agents by the tumors was observed with a multimodal small animal imaging device (FX Pro, America) (Figure 3E).

### *In vitro* Therapeutic Trial

#### Calcein-AM/Propidium Iodide Double Stain

To investigate the photothermal response of FA-PEG/CBT@SPION-ICG, a continuous wave NIR 808 nm laser was adopted. Solutions of FA-PEG/CBT@SPION-ICG, FA-PEG@SPION-ICG, and ICG (ICG concentration of 4 μg/ml or iron concentration of 40 μg/ml) were prepared and added to 24-well plates and cultured with HNE-1 cells in medium for 6 h. Then, the cells were washed with PBS, the medium was replaced with fresh medium, and the cells were treated with 808 nm NIR irradiation of 0.98 W/cm^2^ for 1 min or with magnetic hyperthermia (MHT) (193 kHz, 350.4 A) for 10 min. PBS solution undergoing the same irradiation parameters was used as the negative control sample. Twelve hours later, the cells were stained with a Calcein-AM/PI Double Stain Kit (Yeasen, China). Fluorescence images were captured using an autofluorescence imaging system (Thermo, America) (Figure 4A). The areas of living and dead cells were measured by ImageJ software, and cell mortality (R) was estimated using the following formula.

$${\text{R}=\text{S}}_{\left(\text{D}\right)}{\text{/S}}_{\left(\text{Total}\right)}\times {100\text{\% where S}}_{\left(\text{D}\right)}{\text{ represents the area of dead cells and S}}_{\left(\text{Total}\right)}\text{ represents the area of all cells in the field of vision}\text{.}$$

#### ROS Detection

HNE-1 cells were plated into 24-well plates at 1.4 × 10^5^ cells per well and incubated with 1 ml of complete medium. Agents specific for each group were added to the plate at a concentration of 5 μg/ml and cultured for 6 h. For the PDT test, 1 mM fluorescent dye (DCFH-DA) was added to each well, with NIR irradiation of 0.98 W/cm^2^ for 1 min being applied to the corresponding wells. As the control sample, PBS was irradiated with the same irradiation parameters as above. After continued culture for 2 h, the production of ROS was observed under a fluorescence microscope (Thermo, America) (Figure 4B). We used ImageJ software for general quantitative analysis.

#### Western Blot

According to the requirements of each group, cells were treated with different agents containing 4 μg/ml ICG or 40 μg/ml iron. After laser irradiation at 0.98 W/cm^2^ for 1 min or heat treatment with MHT at 193 kHz, 350.4 A for 10 min, the cells were collected and the total proteins were extracted from each group. Then, the expression of heat shock protein 70 (Hsp70), caspase-3, cleaved caspase-3, PARP, and cleaved PARP was assessed by western blot. β-Actin was adopted as an internal reference protein (Figure 4C).

### *In vivo* Therapeutic Trial

HNE-1 cells (8 × 10^6^/ml) were collected in serum-free medium (200 μl) and subcutaneously injected into the right backs of female BALB/c nude mice. After 1 week, the nude mice bearing HNE-1 tumors with a volume of approximately 50 mm^3^ were randomly divided into seven groups and injected with PBS, FA-PEG@SPION-ICG, or FA-PEG/CBT@SPION-ICG (240 μg/ml ICG, 150 μl for each injection, for a total of four injections) intravenously for 18 consecutive days. Eight hours after each tail vein injection, the mice were treated with a 0.98 W/cm^2^ laser for 5 min or a 193 kHz, 350.4 A magnetic field for 12 min according to the requirements of each group. During this period, the tumor volumes of nude mice were measured every 3 days.

Moreover, we obtained images of the mice before treatment and 2 days after treatment for comparison (Figure 5A). The immediate surface temperature of the tumors after treatment was measured by an infrared thermal imager (DALI, China) 8 h after injection (Figure 5B).

At the end of the experiment, the nude mice were sacrificed by spinal dislocation, and the tumors were harvested and weighed. The tumor inhibition rate R (%) was calculated according to the following formula (Figure 5C).

R (%) = (V_c_-V_E_)/V_c_ × 100%. The average tumor volume in the control group (PBS group) was represented by Vc, while the average tumor volume in the experimental group was represented by V_E_.

The expression of Hsp70 in cancer tissues was further assessed by immunohistochemistry (Figure 5D).

Subsequently, to assess the biosafety of FA-PEG/CBT@SPION-ICG, a CCK-8 assay was adopted, and the organs were histologically examined by hematoxylin and eosin (H&E) staining.

### *In vitro* Cytotoxicity Assay

A CCK-8 assay was used to determine the cytotoxicity of the NPs at different concentrations treated in different ways. HNE-1 cells were seeded in 96-well plates with complete culture medium. Next, they were treated with PBS, FA-PEG@SPION-ICG, or FA-PEG/CBT@SPION-ICG at ICG concentrations of 1, 2, 3, 4, or 5 g/ml for 6 h, with or without laser irradiation (0.98 W/cm^2^ for 1 min) or a magnetic field (193 kHz, 350.4 A for 10 min). To determine HNE-1 cell viability, a cytotoxicity assay was carried out by using a CCK-8 kit (Beyotime, China). The optical density (OD) value was obtained using a microplate reader (Thermo Fisher, America) at 450 nm to determine cell viability (Figure 6A).

### *In vivo* Cytotoxicity Assay

As described before, HNE-1 cells (8 × 10^6^/ml) were collected in serum-free medium (200 μl) and subcutaneously injected into the right backs of female BALB/c nude mice. Next, three tumor-bearing nude mice were injected individually with PBS, FA-PEG@SPION-ICG, or FA-PEG/CBT@SPION-ICG (240 μg/ml ICG, 150 μl for each injection) *via* the caudal vein when the tumor volume reached 200 mm^3^, and the injection was performed once. After 3 days of nanomedication treatment, the mice were sacrificed, and tissues such as the heart, liver, spleen, lung, kidney, and tumor were immediately excised and fixed in tissue glue. Subsequently, the tissues were sectioned into 8 μm thick slices and stained with H&E according to standard clinical pathology protocols. The stained sections were examined by traditional optical microscopy (Leica, Germany) (Figure 6B).

## Results and Discussion

### Characterization of FA-PEG/CBT@SPION-ICG and the Other Key Products

Hydrophilic, aldehyde sodium alginate coated Fe3O4 MNPs (ASA@SPION) were prepared by using previous method reported by our team. Moreover, MNPs coated with aldehydes and carboxylic acid groups were can be robustly functionalized with molecule such as therapeutic drugs, targeting ligands. Next, FA targeting group (FA-PEG-NH_2_) was conjugated with aldehyde on surface of ASA@SPION *via* Schiff base reaction and fallowed by reduction then obtained a FA-PEG@SPION (Bejjanki et al., 2021). ICG has the dual capabilities and can be used as an agent for PTT as well as PDT. Hence, a key functional ICG-amine fragment was conjugated to carboxylic group of FA-PEG@SPION *via* acid amide coupling reaction with EDC/NHS. The efficiency of ICG conjugation was measured through UV absorption spectrum. The conjugation efficiency of FA-PEG@SPION with ICG-NH2 was found to be 60%.

As a key part of synthesizing the final product, the aggregating polypeptide Cys (StBu)-Lys-CBT is crucial to the successful synthesis of the final product FA-PEG/CBT@SPION-ICG. Cys (StBu)-Lys-CBT was further confirmed by NMR as well as mass spectroscopy. There is a peak at approximately m/z value 467.23 in the mass spectrum of Cys (StBu)-Lys-CBT (Supplementary Figure S3), which is consistent with the ideal chemical structural model we built. The disulfide bond (-S-S-) marked with the blue box is the key functional group for the reaction of Cys (StBu)-Lys-CBT with GSH (Supplementary Figure S4).

**FIGURE 2:**
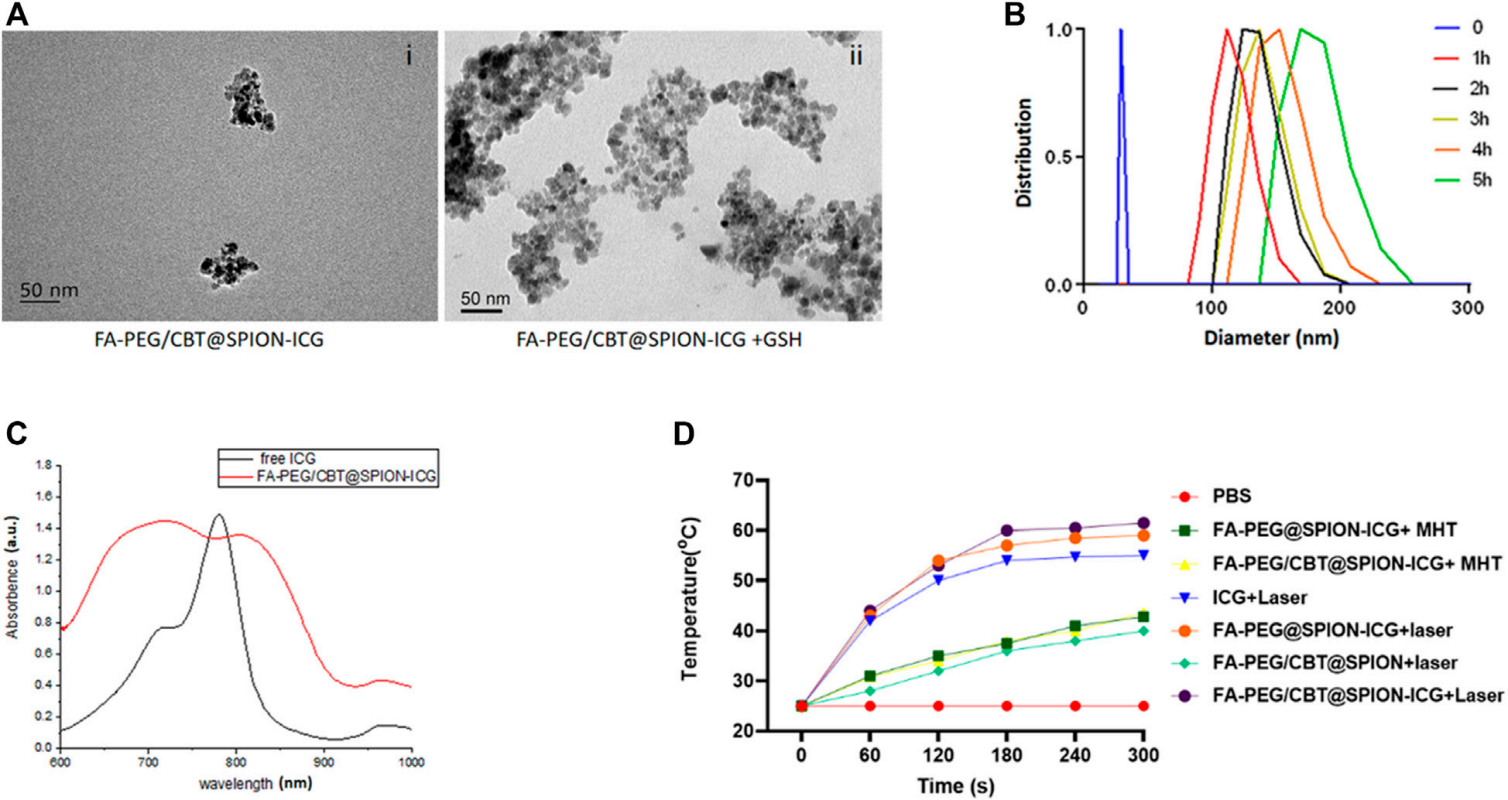
Characterization of FA-PEG@SPION-ICG. TEM images of FA-PEG/CBT@SPION-ICG (i) and FA-PEG/CBT@SPION-ICG after being treated by GSH (ii). DLS diagram of FA-PEG/CBT@SPION-ICG after being treated by GSH **(B)**. UV absorption spectrum of free ICG and FA-PEG/CBT@SPION-ICG **(C)**. *In vitro* temperature profile **(D)**.

To confirm the aggregation ability of Cys (StBu)-Lys-CBT induced by GSH, the Cys (StBu)-Lys-CBT solution was treated with GSH and then the product was detected by high-resolution matrix-assisted laser desorption/ionization mass spectrometry (HR-MALDI/MS) to obtain the molecular weight of the aggregated product. The ion peak in Supplementary Figure S5 (m/z = 819.57) corresponded to the ring dimer model C_34_H_38_N_10_O_4_S_4_ (m/z = 778.2) (Supplementary Figure S6); therefore, the molecular weight deviation between them may be caused by the attachment of potassium (M_[K]_ = 39) and hydrogen (M_[H]_ = 1). Another ion peak m/z = 1,184.75 corresponded to the non-ring trimer model C_51_H_60_N_16_O_6_S_6_ (M = 1,184.32). As observed in Figure 2A, the diameter of FA-PEG/CBT@SPION-ICG was approximately 50 nm through TEM. However, after treatment with GSH, FA-PEG/CBT@SPION-ICG formed larger clusters with diameters of approximately 200 nm.

Finally, we conjugated the NPs with intracellular aggregation peptide Cys (StBu)-Lys-CBT. The primary amine of lysine in Cys (StBu)-Lys-CBT has easily make an amide bond formation with carboxylic acid. So, we next reacted EDC/NHS with PEG/@SPION-ICG, added the Cys (StBu)-Lys-CBT later and obtained FA-PEG/CBT@SPION-ICG.

To further confirm that GSH can induce the condensation and aggregation of FA-PEG/CBT@SPION-ICG by reduction, the NP complex was dispersed in 1 ml of PBS with an ICG concentration of 100 μg/ml. The solution was evenly divided into two parts: one part was used for the GSH-mediated reduction reaction as the experimental group, and the other part was used as the control group without GSH. In the experimental group, 1 mM GSH was added to the suspension and incubated at 37°C for 5 h. During the incubation period, the hydrodynamic diameter of the NPs was measured every hour. The diameter of the NPs mediated by GSH in the experimental group increased over time, from 30 nm at the beginning to 200 nm within 5 h (Figure 2B). Taken together, these results suggested that FA-PEG/CBT@SPION-ICG has good aggregation ability and is responsive to GSH.

MNP have been used as nanocarriers and due to its carboxyl function it can be easily functionalize with different moieties (i.e., targeting, imaging, and therapeutics agents). The functionalization of nanoparticles was confirmed through Fourier transform infrared spectrometer (FTIR) and UV absorption spectrum. The IR spectra of ASA-MNP, FA, Cys (StBu)-Lys-CBT, and FA-PEG/CBT@SPION-ICG are shown in Supplementary Figure S8. The absorption bands at 600-650 cm^−1^ (purple line) in Spectra indicated characteristics of the Fe-O bond of Fe_3_O_4_. Fe-O bonds in FA-PEG/CBT@SPION-ICG has slightly shifted in the range of 580–600 cm^−1^ (violet) in FA-PEG/CBT@SPION-ICG indicate the existence of the Fe_3_O_4_ nanoparticles in FA-PEG/CBT@SPION-ICG.

The folic acid has free carboxylic group and stretching vibrations of C=O observed at 1,730 cm^−1^ (red line) due to free carboxylate. While the broad band between 3,600 and 3,200 cm^−1^ is attributed to O−H and N−H vibrations, respectively. Also, the FTIR spectrum of FA-PEG/CBT@SPION-ICG conjugates contained the characteristic bands of FA and indicated that folic acid was successfully conjugated to the NPs. Farther, the peak at 1,600–1,720 cm^−1^ (pink line) in Cys (StBu)-Lys-CBT represent to the C=N and C=O of amide in benzothiazole. Stretching frequency of C=N and C=O of amide of Cys (StBu)-Lys-CBT in nanoparticles also appear in same region with slight shift (blue line) in the spectrum and clearly indicated the functionalization of peptide.

From Figure 2C, UV-visible spectroscopy was showed the absorption at 600–900 cm^−1^ for ICG and indicated in red, and the FA-PEG/CBT@SPION-ICG also had absorption at the same place and it can prove the ICG to the ASA core.

Additionally, the photothermal efficiency of Nanoparticles were studied by recording the temperature profile with 808 nm laser irradiation of 0.98 W/cm^2^. With NIR, thermal images of FA-PEG/CBT@SPION-ICG was found to be much greater than that of the other suspensions after treatment with NIR or a magnetic field, reaching 60°C after 5 min of exposure to the laser. Here, MHT was selected as the treatment in contrast to photohyperthermia. From the results, we can conclude that neither FA-PEG/CBT@SPION-ICG nor FA-PEG@SPION-ICG after MHT treatment at 193 kHz, 350.4 A can reach 50°C in 5 min. Concerning PTT, FA-PEG/CBT@SPION exposed to the laser can reach a temperature no greater than 40°C in the 5th minute. This showed that the absence of the photosensitizer ICG produced lower temperatures (Figure 2D). Similar results will be further demonstrated *in vitro* and *in vivo*.

### Cellular Uptake of the NPs

**FIGURE 3:**
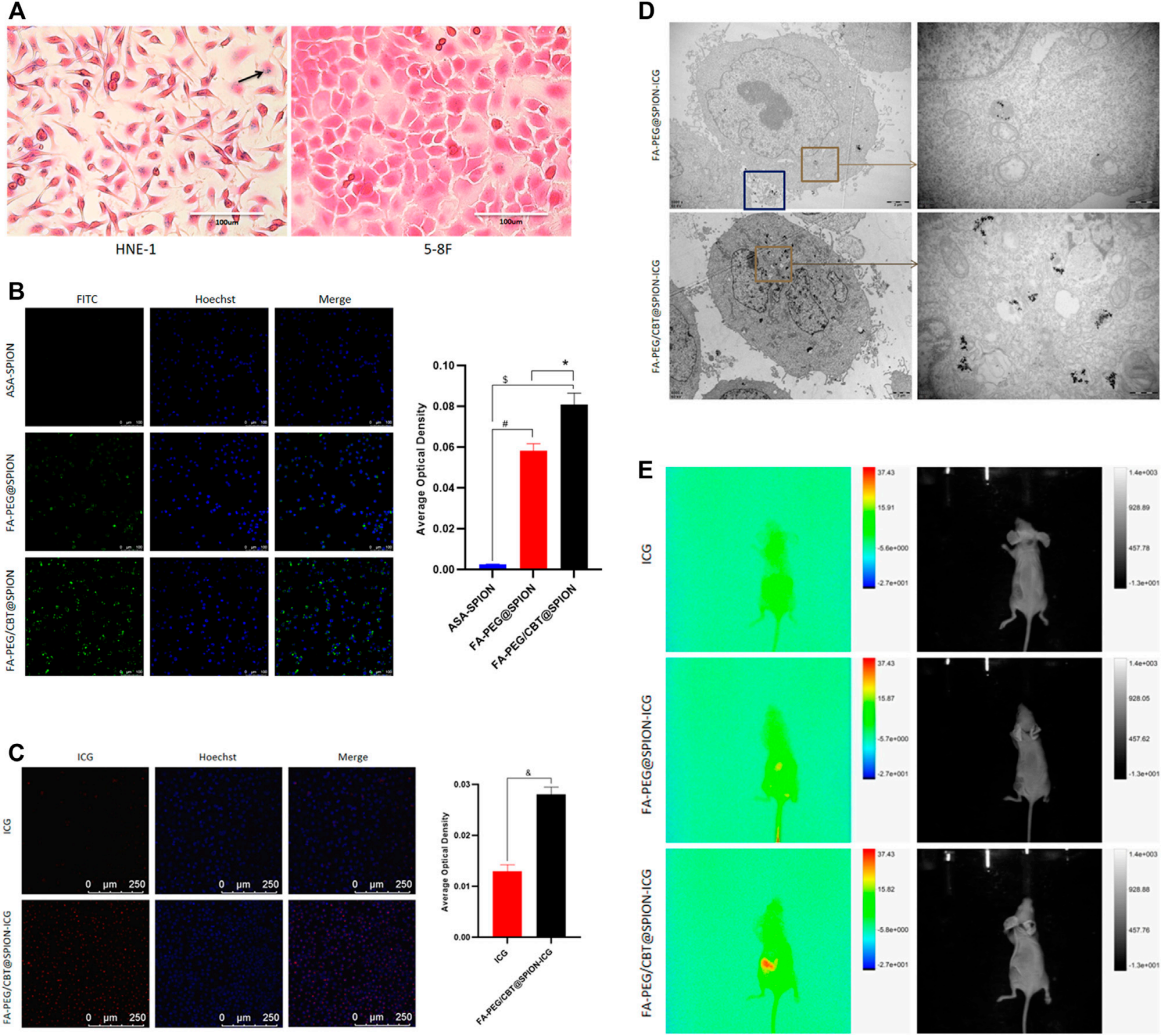
Prussian blue staining **(A)** and Hoechst staining **(B) (C)** of different formations under various conditions. #Stands for *p* < 0.05 ASA@SPION versus FA-PEG@SPION. $ Stands for *p* < 0.05 ASA@SPION versus FA-PEG/CBT@SPION. * Stands for *p* < 0.05 FA-PEG@SPION versus FA-PEG/CBT@SPION. & Stands for *p* < 0.05 ICG versus FA-PEG/CBT@SPION-ICG. TEM results of HNE-1 cells after cellular uptake of FA-PEG@SPION-ICG and FA-PEG/CBT@SPION-ICG **(D)**. Multimodal small animal imaging after injection of different formations at 8 h **(E)**.

To validate the targeting ability of the nanodrug to tumor cells, the FA-loaded intermediate FA-PEG/CBT@SPION was taken up by NPC cells and examined by Prussian staining. Given that HNE-1 cells have been confirmed to have strong positive expression of the FR according to our previous study^[26]^, HNE-1 cells were used as an NPC model *in vitro*. FR-negative NPC 5–8 F cells were used as a control. As shown in Figure 3A, trivalent iron was dyed blue, as the black arrow shows, and the nuclei and other tissues were dyed pink. After incubation for 6 h, clusters of blue granules (FA-PEG/CBT@SPION) could be observed in the cytoplasm of the FR-positive HNE-1 cells, and no blue granules could be observed in the FR-negative 5–8 F cells. This result suggested that FA-PEG/CBT@SPION have a targeted ability for FR-positive HNE-1 cells.

To further verify the tumor-targeting ability of the NPs, we compared the cellular uptake of ASA-SPION, FA-PEG@SPION, and FA-PEG/CBT@SPION in HNE-1 cells using Hoechst staining. Hoechst dye can penetrate the cell membrane and display bright blue fluorescence in the nucleus, while the NPs marked with FITC are green. As shown in Figure 3B, the average optical density (AOD) value of ASA-SPION was 0.002 ± 0.0002, that of FA-PEG@SPION was 0.058 ± 0.003, and that of FA-PEG/CBT@SPION was 0.081 ± 0.006. FA-PEG@SPION and FA-PEG/CBT@SPION showed considerably more absorption, with 29 times and 40.5 times higher AOD values than that of the ASA-SPION group, respectively (*p* < 0.05). The results indicate that the FA-conjugated NPs are capable of promoting the uptake of loaded drugs, which is consistent with the Prussian staining results. In addition, the cellular uptake of FA-PEG@SPION was less than that of FA-PEG/CBT@SPION. This may be due to the lack of Cys (StBu)-Lys-CBT, which serves the function of intracellular aggregation. Herein, the disulfide bonds in Cys (StBu)-Lys-CBT were reduced by GSH in tumor cells, leading nanoparticles become larger, reducing leakage of the NPs and increasing the concentration of NPs in the tumor site.

Subsequently, to prove the uptake superiority of the final product FA-PEG/CBT@SPION-ICG with the aggregating peptide Cys (StBu)-Lys-CBT more intuitively, HNE-1 cells were immobilized and observed by TEM, with FA-PEG@SPION-ICG serving as the control group. As shown in Figure 3D a large amount of FA-PEG/CBT@SPION-ICG aggregates could be observed in the cytoplasm. FA-PEG@SPION-ICG particles were very small in the cytoplasm, with many particles present at the edge of the cell that seemed to be swallowed by phagocytic bodies and ejected from the cell. This may be because the aggregation peptide Cys (StBu)-Lys-CBT can contribute to NP uptake, supporting the above results.

Most importantly, we observed the uptake of the final product FA-PEG/CBT@SPION-ICG, with free ICG as the control. As shown in Figure 3C, after the same incubation time, red fluorescence could be observed in the FA-PEG/CBT@SPION-ICG group, with an AOD value much higher than that of the free ICG group (*p* < 0.05). This result reflects an interesting concept; that free ICG may be more difficult to be taken up by than the nanobased ICG. This result may be closely related to the targeting effects of FA or the aggregation polypeptide Cys (StBu)-Lys-CBT. Therefore, we next set out to determine whether similar results will appear *in vivo*.

### *In vivo* Distribution

To further investigate the absorption and distribution of the NPs *in vivo*, nude mice were intravenously injected with free ICG, FA-PEG@SPION-ICG, and FA-PEG/CBT@SPION-ICG, reared for 8 h and then observed with a small animal imaging device. The highest amount of FA-PEG/CBT@SPION-ICG could be observed at the tumor location, followed by FA-PEG@SPION-ICG. No significant fluorescence signal was observed in the tumor site in the free ICG group (Figure 3E). These results again reflect that the aggregation peptide Cys (StBu)-Lys-CBT as well as FA targeting ligand can promote the uptake of NPs by tumor cells. Moreover, nanobased ICG could be taken to a greater extent by tumor tissues than free ICG over 8 h, which was consistent with the *in vitro* uptake. Previous studies have reported that the encapsulation of ICG into the lipid coating of superparamagnetic iron oxide (SPIO) NPs results in higher photostability than free ICG due to protection from degradation (Ma et al., 2013). This provides an additional experimental basis for the analysis of our results. The connection of ICG with other functional aptamers can improve its water stability, thus effectively avoiding the rapid decomposition and removal of ICG. FA-PEG/CBT@SPION-ICG has shown higher uptake than FA-PEG@SPION-ICG, both having FA targeting and former having additional aggregating ability which might be increase the concentration in the cytoplasm. FA-PEG/CBT@SPION-ICG may have very large potential for the translation of basic research to the clinical treatment of various tumors.

### *In vitro* Antitumor Effects of FA-PEG/CBT@SPION-ICG

#### Live/Dead Cell Staining

**FIGURE 4:**
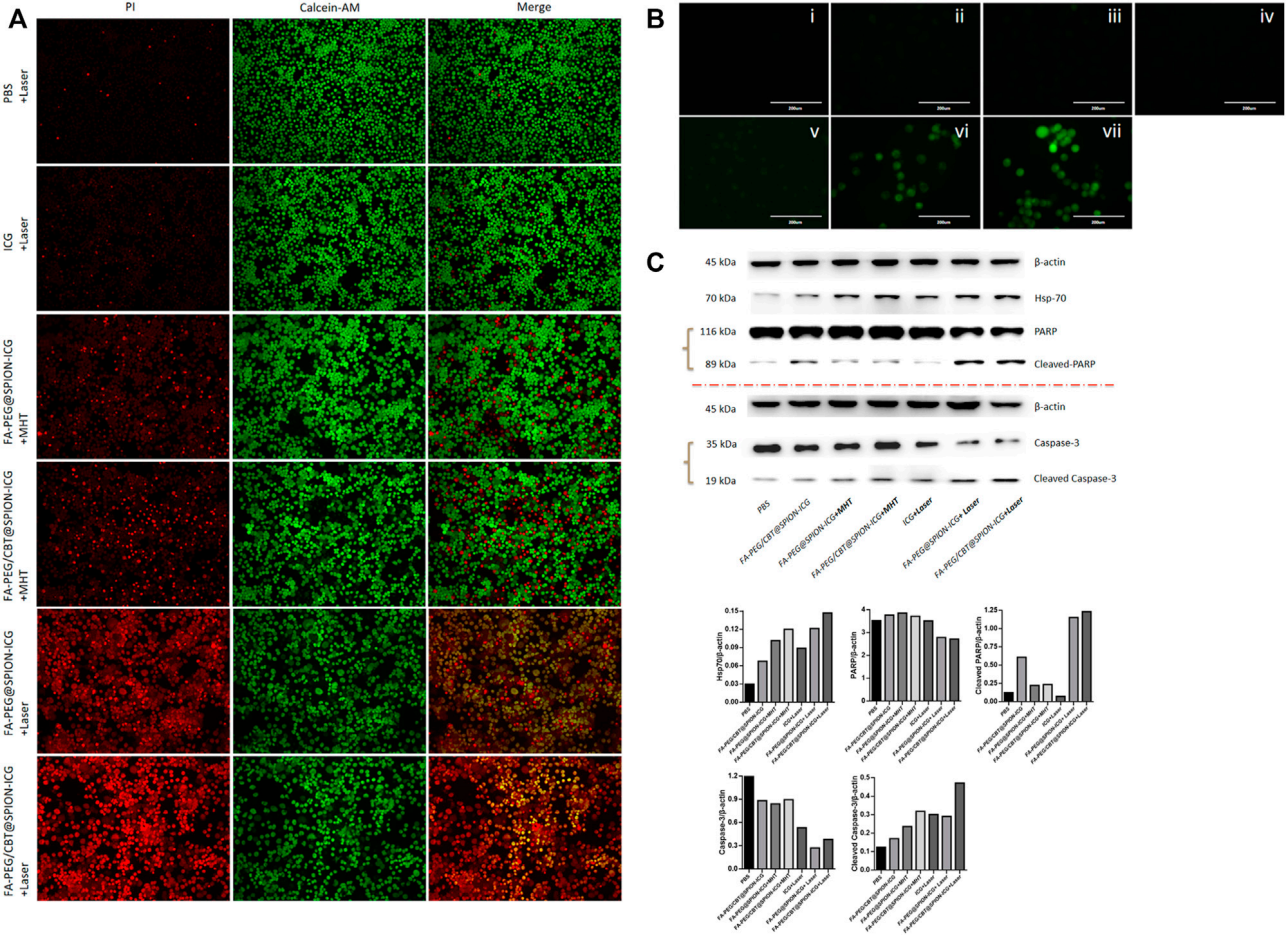
Calcein-AM/PI live/dead cell staining (10 ×) **(A)**, ROS detected by DCFH-DA staining **(B)**, and western blot analysis **(C)** of different formations under various conditions. In **B**, i is PBS, ii is FA-PEG@SPION-ICG, iii is FA-PEG/CBT@SPION-ICG, iv is PBS + Laser, v is ICG + Laser, vi is FA-PEG@SPION-ICG + Laser, and vii is FA-PEG/CBT@SPION-ICG + Laser.

As shown in Figure 4A, the laser groups emitted more red fluorescence than the other groups. Among them, the mortality of the cells after treatment with FA-PEG@SPION-ICG and the laser was 69.8% according to the formula above, and the mortality of the cells after treatment with FA-PEG/CBT@SPION-ICG and the laser was 86.5%. This difference may be due to the application of the aggregation peptide Cys (StBu)-Lys-CBT. This peptide caused the NPs to polymerize in cells, which can enhance the photosensitivity of the NPs and improve antitumor efficiency. However, distribution of the red fluorescence signals was reduced in the MHT-treated cells. Both FA-PEG@SPION-ICG and FA-PEG/CBT@SPION-ICG with MHT treatment exhibited low cell mortality, 13.4 and 16.6%, respectively. In comparison, MHT-treated NPs at the same concentration of iron and ICG may be capable of exerting therapeutic effects that are not as effective as NIR. We know that iron-core NPs are modified by multiple functional molecules, and they may weaken the intrinsic magnetic responsiveness of NPs and limit heat production. This could explain why the therapeutic effects of the MHT-treated groups was not as good as that of the NIR-treated groups. More notably, at the same concentration of ICG, a small amount of red fluorescence could be observed in the NIR-treated free ICG group. This reveals that nanobased ICG can achieve higher treatment efficiency as a photosensitizer than free ICG. The reason for this result may be closely related to the targeted uptake by tumor cells, and the cellular accumulation of nanodrugs at a high concentration can contribute to efficient PTT.

#### Detection of ROS in Cells

Furthermore, to explore the mechanism of cell death *via* photodynamics and determine the potential mechanism, we carried out an experiment with an ICG concentration of 5 μg/ml to detect the production of ROS after treatment with NIR. As a photosensitive material, ICG can produce ROS by PDT under irradiation with NIR light. According to Figure 4B, green fluorescence could be observed only in the free ICG, FA-PEG@SPION-ICG, and FA-PEG/CBT@SPION-ICG groups. Among them, the NIR-treated free ICG group showed the weakest fluorescence with an AOD value of 0.012, which may be related to the degradability of ICG itself and the low uptake capacity of free ICG by HNE-1 cells. FA-PEG@SPION-ICG and FA-PEG/CBT@SPION-ICG gave AOD values of 0.032 and 0.051, respectively. This demonstrates that in addition to thermal damage, FA-PEG/CBT@SPION-ICG exposed to NIR can also function through photodynamic therapeutic mechanisms *via* the induction of ROS. Moreover, the difference in the AOD results between FA-PEG@SPION-ICG and FA-PEG/CBT@SPION-ICG suggests that Cys (StBu)-Lys-CBT can enhance the photo toxicity *via* GSH catalyzed intracellular aggregation.

#### Western Blot

As a sensitive stress protein, Hsp70 can be stimulated and its expression significantly increased in the cytoplasm when the ambient temperature rises. According to the western blot analysis (Figure 4C), PTT could induce Hsp70. Caspase-3 and PARP activation assays confirmed apoptosis as the cell death modality observed in our study. Hsp70 was detected in all NIR-treated and MHT-treated groups. Regarding apoptosis indicators, only FA-PEG@SPION-ICG and FA-PEG/CBT@SPION-ICG exposed to NIR showed caspase-3 activation and PARP activation at the same time. This discrepancy in protein expression may be related to the temperature threshold of the tumor. When the tumor tissue was within the threshold, there was no significant correlation between the expression of Hsp70 and the temperature of the tumor. If the temperature exceeded the threshold, as in this experiment, then, the higher the temperature was, the more Hsp70 was expressed. We can conclude that among all seven groups, the final product FA-PEG/CBT@SPION-ICG combined with laser treatment can have the most lethal effect on tumor cells.

### Therapeutic Effects *In vivo*

**FIGURE 5:**
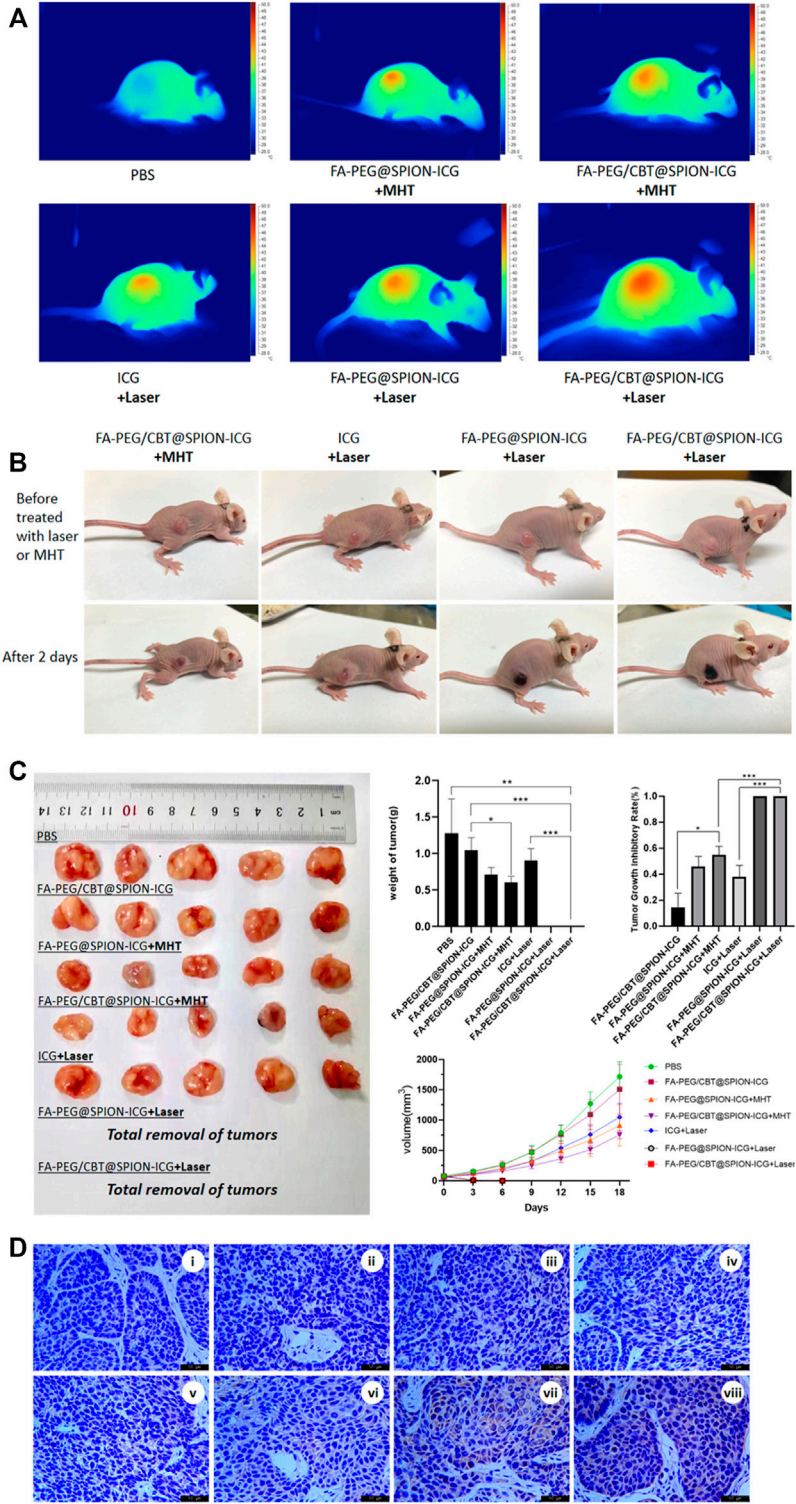
Thermal imaging of the mice after treatment with different formulations with NIR or MHT **(A)**. Imaging was performed before and on day 2 after the different treatments **(B)**. Tissues dissected from nude mice, mass histogram of the tumor weight, mass histogram of the tumor growth inhibition rate, and a line graph of tumor tissue volume over time **(C)** (N = 5) (*) *p* < 0.05 (**) *p* < 0.01 (***) *p* < 0.001. Immunohistochemical analysis of Hsp70 expression in different groups **(D)**. **Figure 4D**, i is PBS, ii is PBS + Laser, iii is FA-PEG/CBT@SPION-ICG, iv is FA-PEG@SPION-ICG + MHT, v is FA-PEG/CBT@SPION-ICG + MHT, vi is free ICG + Laser, vii is FA-PEG@SPION-ICG + Laser, and viii is FA-PEG/CBT@SPION-ICG + Laser.

As shown in Figure 5A, the tumor surface temperature of the MHT-treated groups was 42–44°C, while the tumor surface temperature in the NIR-treated groups reached up to 46.2°C after 1 min of irradiation. After laser or magnetic hyperthermia treatment and observation for 5 min, obviously dilated blood vessels could be seen on the tumor surface in NIR combined with NPs-treated groups, and the surface was slightly dark red. In MHT-treated groups, there was less vasodilation on the surface, and the color was slightly lighter than the former, while there was no obvious change in tumor appearance in other groups. After 2 days of treatment, a slight brown scab occurred on the tumor surface after treatment with FA-PEG/CBT@SPION-ICG exposed to the magnetic field, and an obvious black scab could be observed in the FA-PEG@SPION-ICG and FA-PEG/CBT@SPION-ICG groups exposed to NIR, as shown in Figure 5B. The results confirmed that FA-PEG/CBT@SPION-ICG combined with PTT had a good therapeutic effect on nude mice bearing HNE-1 tumors compared with the MHT-treated groups.

Similarly, as shown in Figure 5C, the tumors in the nude mice treated with FA-PEG@SPION-ICG and FA-PEG/CBT@SPION-ICG exposed to NIR showed remarkably limited growth, with the tumors nearly being eradicated, proving the best antitumor effect. Following MHT treatment, the tumor growth inhibitory rates of the two groups were approximately 47 and 56% for FA-PEG@SPION-ICG and FA-PEG/CBT@SPION-ICG, respectively. Furthermore, when compared to the PBS group, the weights of the tumors in the MHT-treated groups decreased remarkably. This indicates that magnetic hyperthermia indeed has some effects on tumors but these effects are not as effective as laser treatment. In addition, in the NIR-treated groups, it should be noted that because the black scab layer was very deep, it was extremely difficult to dissect and isolate the tumors from the normal tissues, so the tumor tissue was regarded as completely absent.

Immunohistochemical analysis of Hsp70 expression in tumor tissues indicated the highest positive proportion in the FA-PEG@SPION-ICG and FA-PEG/CBT@SPION-ICG exposed to NIR groups (Figure 5D), suggesting that the functional mode in our study was accompanied by the production of Hsp70. The expression of Hsp70 in the NIR-treated groups was much higher than that in the MHT-treated groups. This result and those of the Western blot analysis are well matched.

For the therapeutic efficacy studies *in vivo*, all evidence suggests FA-PEG/CBT@SPION-ICG with NIR treatment can achieve the greatest benefit to suppress tumor development. The mechanisms involved in killing tumors in our study may be related to thermal damage, ROS generation, and apoptotic protein activation. According to the appearance of tumor, the tumor tissues in NIR combined with NPs-treated groups were thermally damaged, and the vascular endothelial cells might be damaged by ROS in the early stage after treatment, resulting in microcirculation disturbance, while the tumor cells were not obviously damaged. Next, the continuous action of ROS and the lack of blood supply led to the production of apoptotic proteins and the damage and death of tumor cells. In addition, there is a research reports that NPs can trigger an immune response in animals that destroys the tumor (Bourquin et al., 2020). When exposed to laser irradiation, tumor cells are exposed to new antigens, which may be recognized by T cells around the tumor and send out signals that can help the immune system produce a certain antitumor response, which provides a possible complement to the mechanism of antitumor effects in our study that deserves further study.

### Cytotoxicity Assay

**FIGURE 6:**
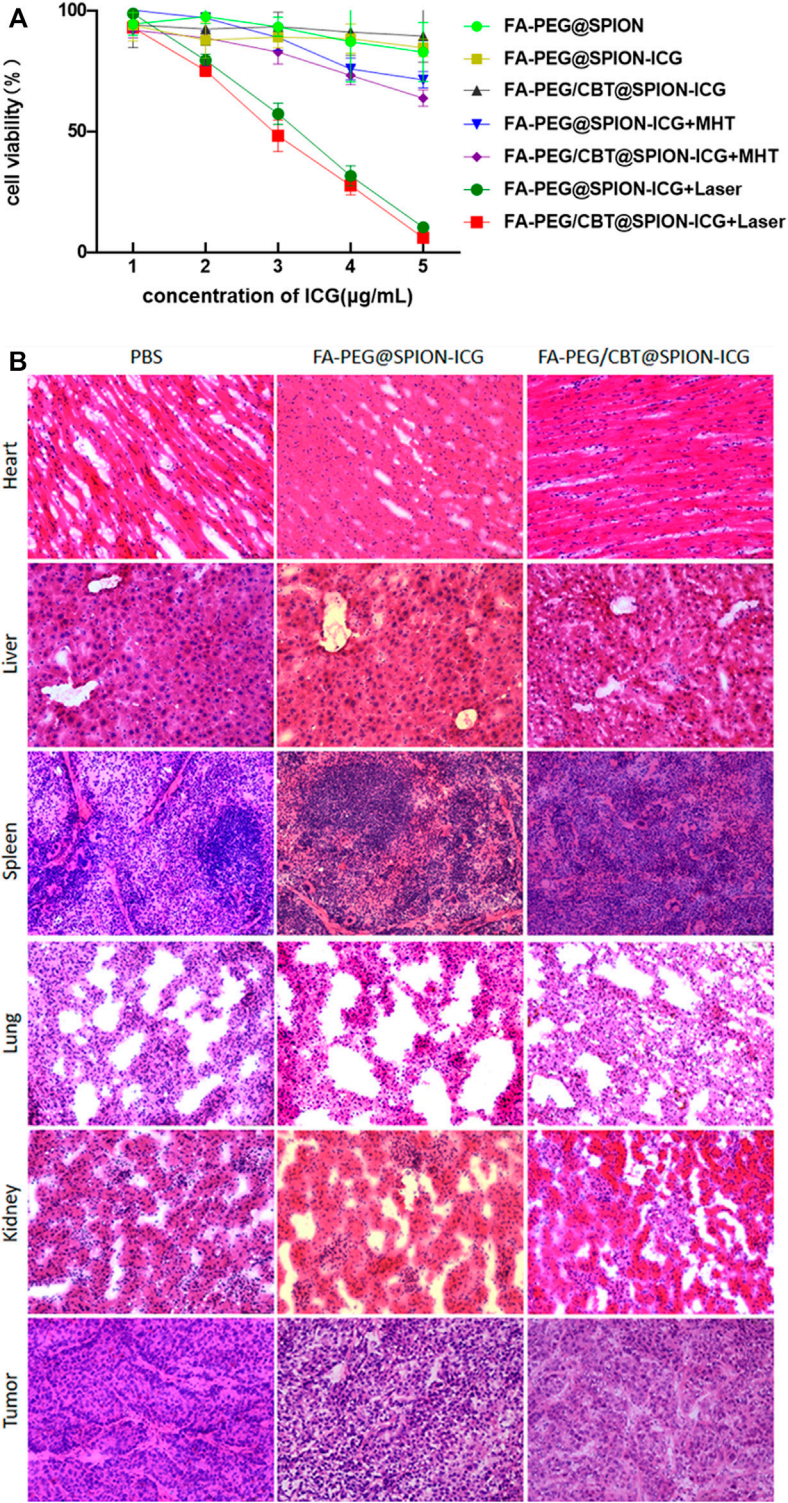
Cell viability detected by CCK-8 assay **(A)**. H&E staining of the heart, liver, spleen, lung, kidney, and tumor tissues of nude mice (× 20) **(B)**.

To investigate the toxic effects to NPC cells of the synthetic drugs without NIR or MHT treatment and explore the therapeutic effects of FA-PEG@SPION-ICG and FA-PEG/CBT@SPION-ICG undergoing PTT and MHT at various ICG concentrations on the treatment of HNE-1 cells, seven groups were analyzed using a CCK-8 assay. As shown in Figure 6A, the cell viability was 89.6 ± 10.8% after FA-PEG/CBT@SPION-ICG treatment at an ICG concentration of 5 μg/ml, showing almost no toxic effects on the tumor cells. However, after exposure to NIR or combined with MHT, the cell viability decreased significantly at the same ICG concentration of 5 μg/ml. The cell viabilities of FA-PEG@SPION-ICG and FA-PEG/CBT@SPION-ICG undergoing MHT were 71.5 ± 3.4, and 63.9% ± 3.3%, respectively. When exposed to NIR, the cell viabilities decreased to 10.5 ± 1.0% and 6.2 ± 0.2%, respectively, showing a significant reduction compared with that of the other groups (*p* < 0.05).

The vital organs were dissected and sectioned for H&E staining. As shown in Figure 6B, there were no visible signs of cell damage in the heart, liver, spleen, or lung tissues, indicating low toxicity to the body. H&E-stained tumor sections also showed little tissue damage, further validating the safety of the final product FA-PEG/CBT@SPION-ICG. This result is consistent with the results of the *in vitro* trials. Therefore, FA-PEG/CBT@SPION-ICG may be a material suitable for NIR imaging and photothermal destruction of NPC, which has good biological safety and is promising strategy for clinical research in the future.

## Conclusion

In summary, the photothermally responsive NPs FA-PEG/CBT@SPION-ICG with FA-targeting and intracellular aggregation abilities were successfully synthesized and characterized using various techniques, such as TEM, ^1^H NMR, and mass spectrometry. The mechanism of cell death is related to the generation of ROS, the production of Hsp70, and the activation of apoptotic proteins (caspase-3, PARP). FA-PEG/CBT@SPION-ICG combined with NIR irradiation has a good therapeutic effect as well as good biological safety and is expected to be a complementary treatment model for HNE-1 NPC.

## Data Availability

The original contributions presented in the study are included in the article/Supplementary Material, further inquiries can be directed to the corresponding author.

## Abbreviations

NPC, nasopharyngeal carcinoma; NIR, near-infrared; PTT, photothermal therapy; PDT, photodynamic therapy; FA, folic acid; FR, folate receptor; ICG, indocyanine green; CBT, 2-cyanobenzothiazole; CBT-Cys, 2‐cyanobenzothiazole‐cysteine; ASA, aldehyde sodium alginate; MNP, magnetic iron oxide nanoparticle; GSH, glutathione; NPs, nanoparticles; MHT, magnetic hyperthermia; SPIONs, superparamagnetic iron oxide nanoparticles; EDC, 1-(3-Dimethylaminopropyl)-3-ethylcarbodiimide; NHS, N-Hydroxysuccinimide; DMSO, dimethyl sulfoxide.
