# Supplementary material for: Synthesis and Photothermal Effects of Intracellular Aggregating Nanodrugs Targeting Nasopharyngeal Carcinoma
Source: Front Bioeng Biotechnol. 2021 Sep 16;9:730925. doi: 10.3389/fbioe.2021.730925 (PMC8481884; doi:10.3389/fbioe.2021.730925)

Figure S1 Proposed reaction scheme for the fabrication of Cys(StBu)-Lys-CBT.

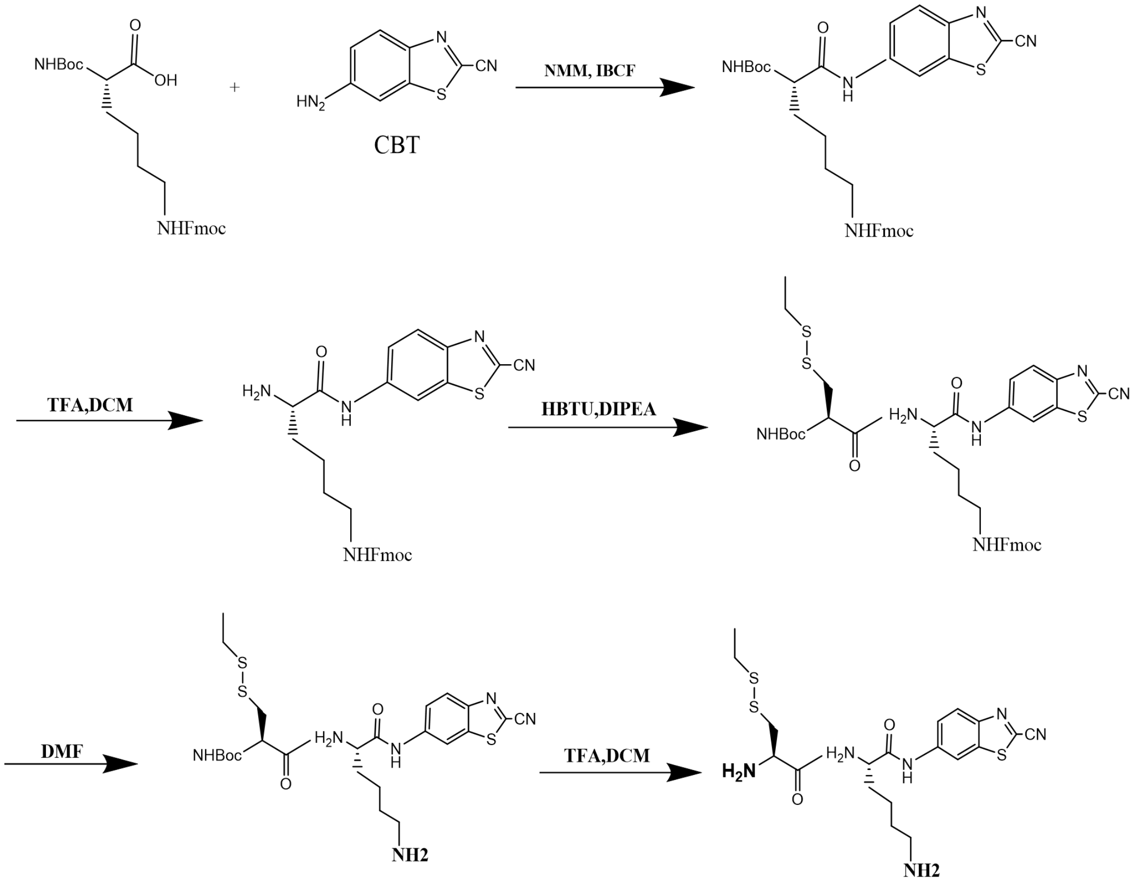

Figure S2 Proposed reaction scheme for the fabrication of FA-PEG/CBT@SPION-ICG.

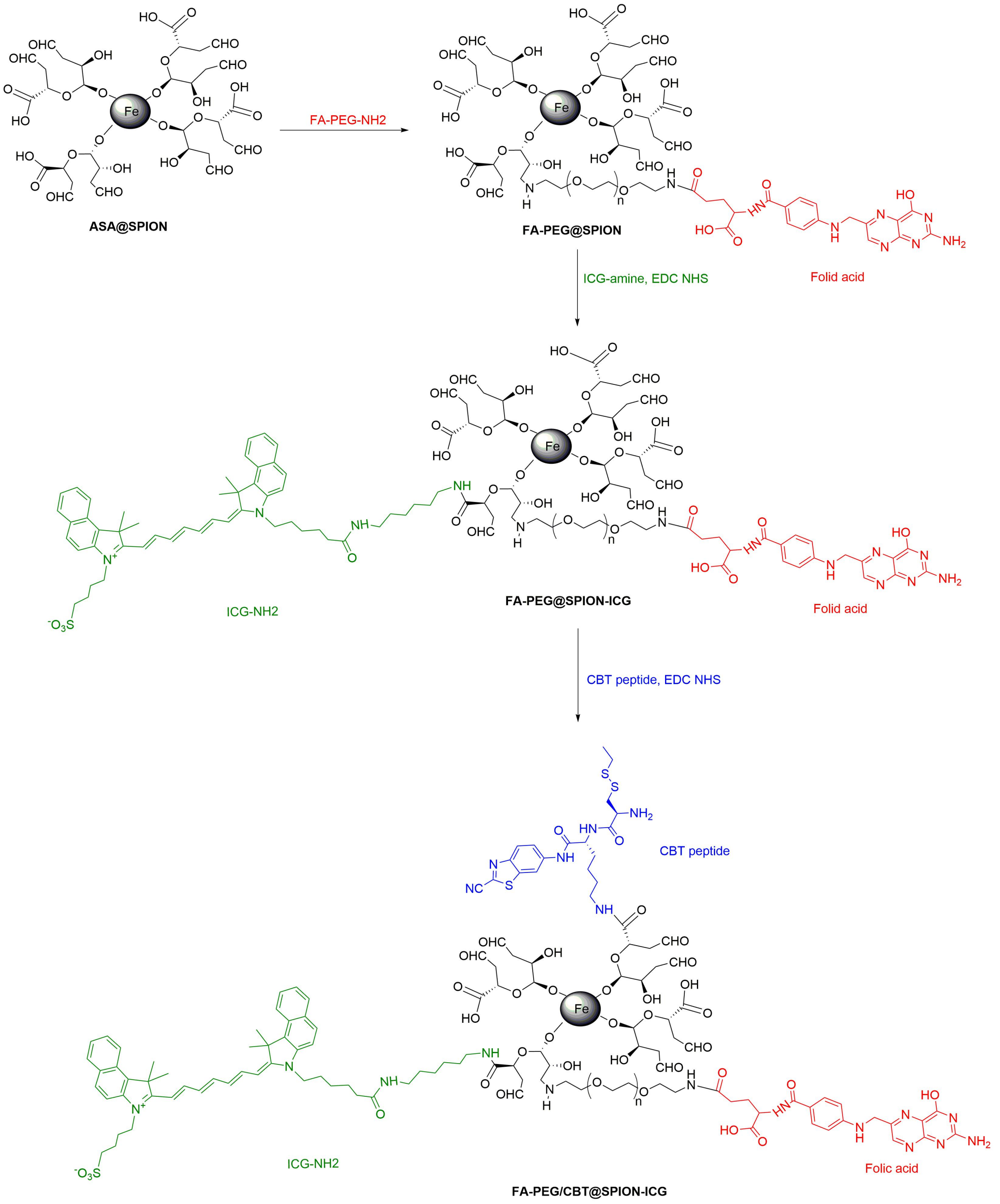

Figure S3 Mass Spectrogram of Cys(StBu)-Lys-CBT.

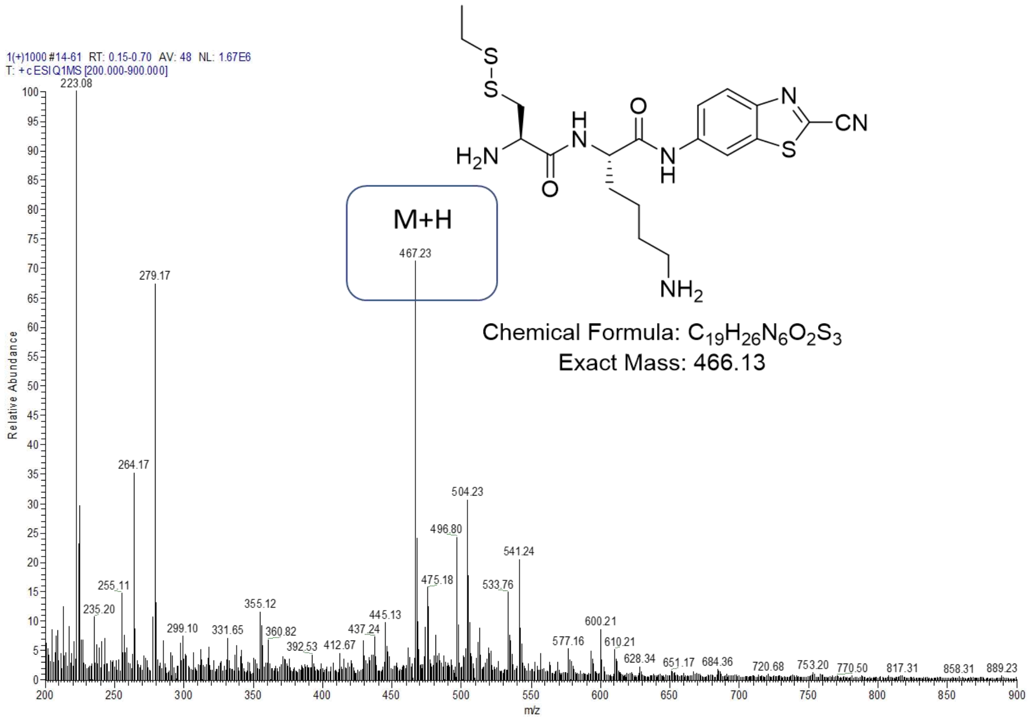

Figure S4 Chemical Structure of Cys(StBu)-Lys-CBT.

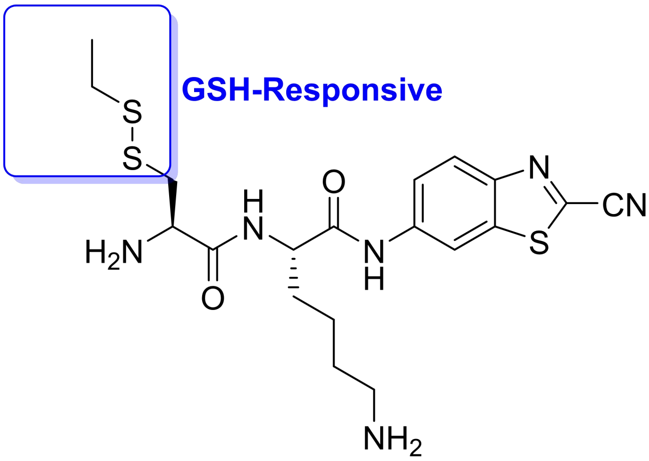

Figure S5 HR-MALDI/MS of Cys(StBu)-Lys-CBT treated by GSH

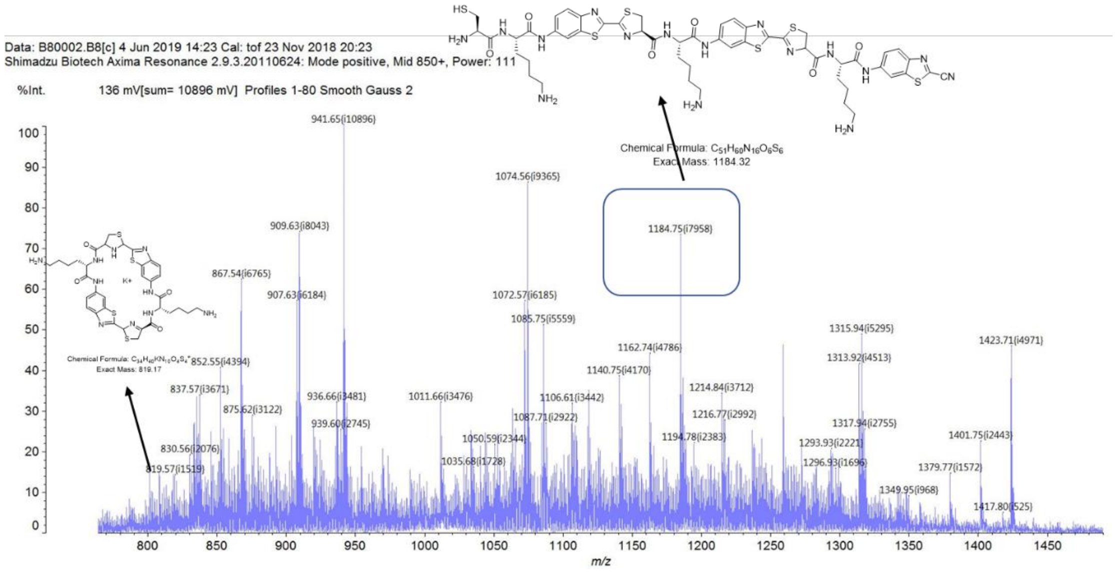

Figure S6 After Cys(StBu)-Lys-CBT was treated by GSH， two kinds of structures were inferred from mass spectrometry ， one of which is ring dimer $C_{34}H_{38}N_{10}O_4S_4$(M=778.2) and the other one is non-ring trimer $C_{51}H_{60}N_{16}O_6S_6$(M=1184.32)

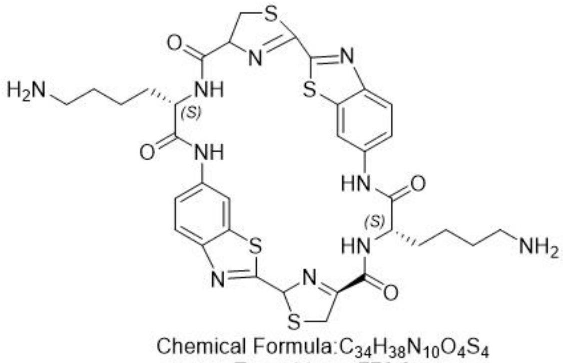

Chemical Formula: $C_{34}H_{38}N_{10}O_4S_4$
Exact Mass: 778.2

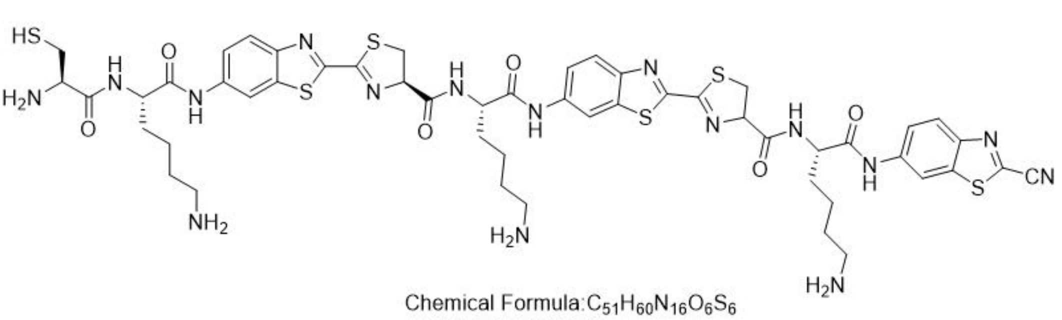

Chemical Formula: $C_{51}H_{60}N_{16}O_6S_6$
Exact Mass: 1184.32

Figure S7 The nuclear magnetic resonance hydrogen spectrum of Cys(StBu)-Lys-CBT

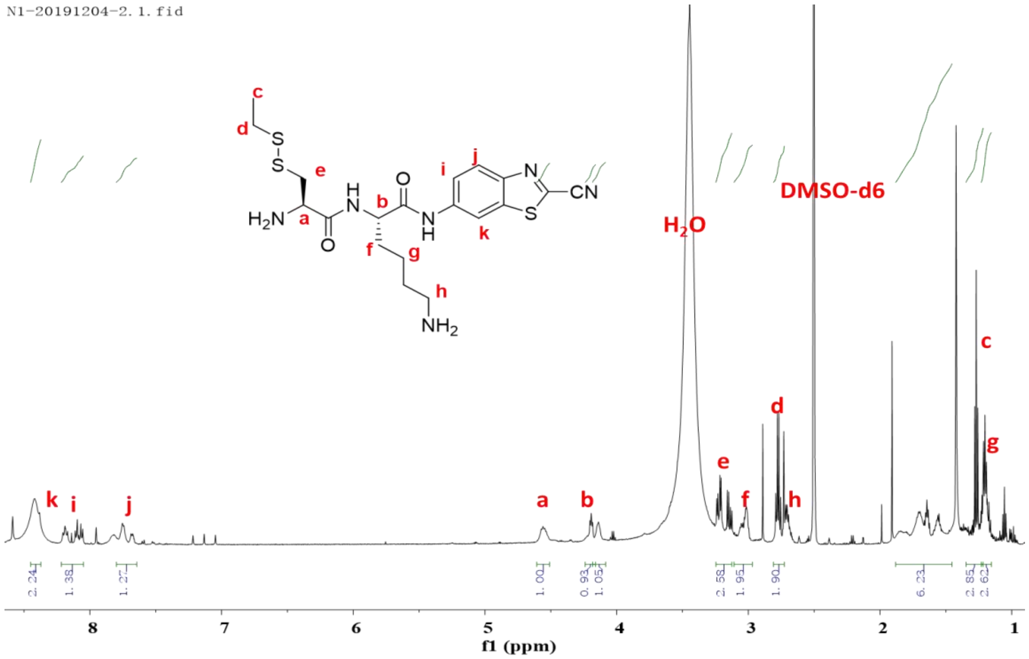

The ^{1}H NMR spectrum supported the chemical structure of Cys(StBu)-Lys-CBT (Figure S6). ^{1}H NMR (DMSO-d6, 500 MHz): 1.23 (t, J = 7.2 Hz, 3 H), 1.35-2.00 (m, 6 H), 2.74-2.80 (m, 2H), 2.90-3.05 (m, 2 H), 3.14 (dd, J = 14.4, 7.0 Hz, 1H), 3.22 (dd, J = 14.4, 5.0 Hz, 2H), 4.20 (t, J = 6.0 Hz, 1 H), 4.50-4.58 (m, 1H), 7.60-7.8 (m, 1 H), 8.1 (dd, 1H, J = 12.2, 9.0 Hz, 1 H), 8.4 (s, 1H). MS (ESI): m/z 467 [(M+H)+].

Figure S8 UV absorption spectrum of different products .

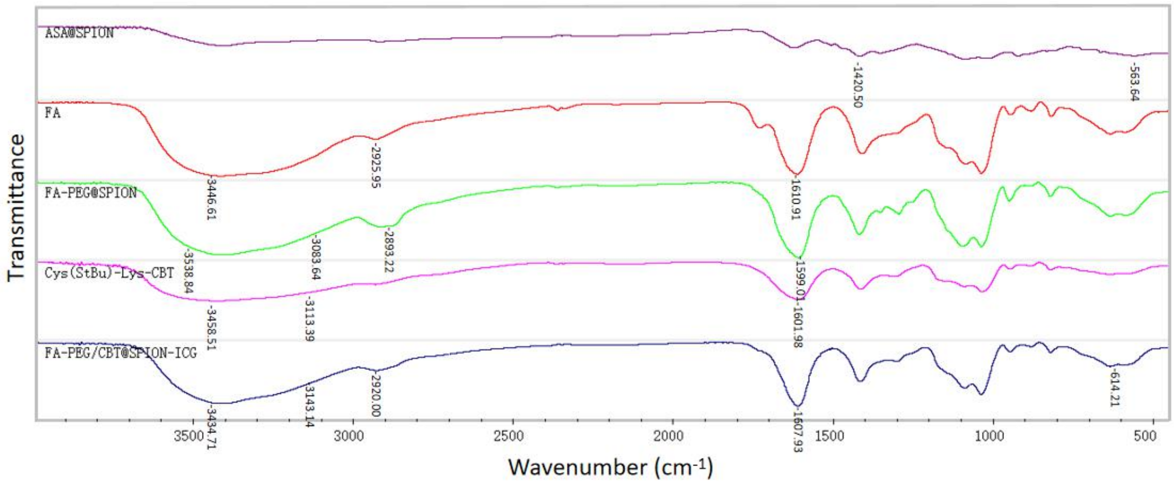

Supplement: Supplementary file 1 [file Image1.PDF]
